# Continued Botanical Exploration Uncovers a New Endemic Species of *Curcuma* subgen. *Hitcheniopsis* (Zingiberaceae) from Central Thailand †

**DOI:** 10.3390/life16101623

**Published:** 2026-09-28

**Authors:** Surapon Saensouk, Piyaporn Saensouk, Thawatphong Boonma, Prasert Ruannakarn, Auemporn Junsongduang, Ahmad Dwi Setyawan

**Affiliations:** 1 Diversity of Family Zingiberaceae and Vascular Plant for Its Applications Research Unit, Walai Rukhavej Botanical Research Institute, Mahasarakham University, Maha Sarakham 44150, Thailand; boonma.thawat@gmail.com; 2 Diversity of Family Zingiberaceae and Vascular Plant for Its Applications Research Unit, Department of Biology, Faculty of Science, Mahasarakham University, Maha Sarakham 44150, Thailand; 3 Department of Educational Research and Development, Faculty of Education, Mahasarakham University, Maha Sarakham 44000, Thailand; prasert.rua@msu.ac.th; 4 Department of Science and Technology, Faculty of Liberal Arts and Science, Roi Et Rajabhat University, Selaphum District, Roi Et 45120, Thailand; a.junsongduang@reru.ac.th; 5 Biodiversity Research Group, Universitas Sebelas Maret, Jl. Ir. Sutami 36A, Surakarta 57126, Indonesia; ahmad@staff.uns.ac.id

**Keywords:** *Curcuma*, *Hitcheniopsis*, limestone, phenetic analysis, Phra Phutthabat, Saraburi, taxonomy

## Abstract

Continued botanical exploration in previously insufficiently surveyed areas of Saraburi Province, Central Thailand, revealed an undescribed member of *Curcuma* subgen. *Hitcheniopsis*. This study aimed to formally describe the taxon, assess its morphological distinctiveness, evaluate its phenetic relationships, and provide a preliminary conservation assessment. Morphological observations were based on living and freshly collected material, herbarium specimens, protologues, and comparative taxonomic sources. Phenetic relationships among 11 taxa were assessed using 34 morphological characters, the Gower coefficient, UPGMA clustering, and principal coordinates analysis (PCoA). The new species, *Curcuma koonmeeana* Saensouk, P.Saensouk & Boonma, is distinguished from morphologically allied taxa by a unique combination of fertile-bract architecture, poorly developed coma bracts, well-exserted flowers, labellum morphology and indumentum, yellow markings, and anther-crest configuration. Phenetic analyses summarized overall morphological relationships among the sampled taxa, with *Curcuma charanii* Boonma & Saensouk showing the greatest Gower similarity to *C. koonmeeana*. The species is currently known from two populations along the same limestone mountain range and is provisionally assessed as Vulnerable (VU D1). Its recognition increases the known native diversity of subgen. *Hitcheniopsis* in Thailand to 31 taxa, comprising 30 species and one natural hybrid. The discovery highlights the continuing value of targeted botanical exploration and the need for habitat and population conservation.

## 1. Introduction

*Curcuma* L. (Zingiberaceae) is one of the largest and most morphologically diverse genera of the ginger family, comprising approximately 180 accepted species distributed mainly throughout tropical and subtropical Asia, with its range extending to northern Australia [1]. The genus includes species of considerable ecological, ornamental, medicinal, and cultural importance and is particularly diverse in mainland Southeast Asia [1,2]. Its remarkable variation in vegetative architecture, inflorescence structure, bract morphology, and floral characters has historically resulted in differing interpretations of infrageneric relationships. Morphological classifications recognized several groups within *Curcuma*, whereas subsequent molecular phylogenetic studies supported a broader circumscription of the genus and its division into three principal subgenera: subgen. *Curcuma*, subgen. *Ecomata* Škorničk. & Šída f., and subgen. *Hitcheniopsis* (Baker) K.Schum [2,3,4].

Among these, *Curcuma* subgen. *Hitcheniopsis* is characterized principally by the absence of epigynous glands and, in most species, by spurless or nearly spurless anthers, together with a suite of inflorescence and floral characters [3,4,5]. Although its circumscription has become clearer following phylogenetic and taxonomic reassessments, morphological variation within the subgenus remains substantial, and species delimitation continues to rely heavily on detailed comparison of vegetative and reproductive characters. Recent studies have continued to document previously unrecognized members of the subgenus in mainland Southeast Asia, particularly in Thailand, indicating that its species diversity and geographical distribution remain incompletely understood [5,6].

Thailand represents a major centre of diversity of *Curcuma*, with species occurring across a wide range of habitats, including limestone formations, sandstone landscapes, deciduous forests, and seasonally dry environments [2,5]. Continued botanical exploration has resulted in the recognition of numerous narrowly distributed and endemic taxa, demonstrating that even areas subjected to previous floristic investigation may retain undocumented diversity. Saraburi Province in Central Thailand is particularly notable for its rich Zingiberaceae flora. Taxonomic exploration in the province has resulted in the description of species such as *Curcuma saraburiensis* Boonma & Saensouk from Phra Phutthabat District [7], while a subsequent province-wide investigation documented 103 species of Zingiberaceae, including 14 taxa endemic to Thailand and 23 species newly recorded for the province, together with the description of *Kaempferia chaveerachiae* Saensouk, P.Saensouk & Boonma [8]. Nevertheless, several mountainous and difficult-to-access localities could not be adequately investigated during that survey, leaving potentially important gaps in botanical coverage.

To address these gaps, additional field investigations were conducted in Saraburi Province in 2025, with particular attention to previously insufficiently explored localities. During these surveys, an unusual population of *Curcuma* subgen. *Hitcheniopsis* was discovered in Phra Phutthabat District. The plants superficially resemble *C. charanii* Boonma & Saensouk, *C. puangpeniae* Boonma & Saensouk, and *C. purpurata* Boonma & Saensouk [9], but detailed examination revealed a distinctive combination of vegetative, inflorescence, and floral characters that could not be accommodated within any currently recognized species. The present study therefore aims to formally describe and illustrate this taxon as *Curcuma koonmeeana* Saensouk, P.Saensouk & Boonma, sp. nov., evaluate its morphological distinctiveness through detailed comparison with allied species, and characterize its overall morphological relationships using quantitative and qualitative phenetic analyses. The taxonomic recognition of *C. koonmeeana* is based primarily on comparative morphological evidence, while the phenetic analyses provide a quantitative overview of its morphological relationships with selected members of subgen. *Hitcheniopsis*.

## 2. Materials and Methods

### 2.1. Field Surveys and Plant Materials

Additional botanical exploration was conducted in Phra Phutthabat District, Saraburi Province, Central Thailand (Figure 1), on 11 August 2025 and 8 August 2026, following the previous province-wide investigation of Zingiberaceae [8]. Fieldwork focused on mountainous localities that had not been examined during the previous survey because of limited accessibility and logistical constraints.

During these surveys, an unusual population of *Curcuma* subgen. *Hitcheniopsis* was discovered in mixed deciduous forest on limestone hills. The taxon is currently known from two populations occurring along the same mountain range. Living plants were photographed in situ before collection, and observations were made on habitat, associated plant species, phenology, population size, and potential threats. The combined population was visually estimated to comprise fewer than 500 mature individuals; however, a complete census of mature individuals was not undertaken.

Morphological observations were based on 30 mature plants from the two known populations. Voucher specimens were prepared following standard herbarium procedures [10] and deposited in the Vascular Plant Herbarium, Mahasarakham University (VMSU), Thailand. The type collection, *T.Boonma SB2581*, was collected on 11 August 2025 from Phra Phutthabat District, Saraburi Province, Central Thailand.

### 2.2. Morphological Observations and Measurements

Morphological characters were examined primarily from living mature plants in the field and from freshly collected flowering and fruiting material. Vegetative structures were measured using a ruler and vernier caliper (Mitutoyo Corporation, Kawasaki, Japan), whereas floral structures were dissected and examined under a stereomicroscope (Stemi 2000-C; Zeiss, Oberkochen, Germany). Floral measurements were taken from freshly opened flowers to minimize changes in size, shape, colour, and posture resulting from senescence or preservation.

The characters examined included habit, rhizome, rhizome branches, roots and root tubers, leafy shoot, bladeless sheaths, leaf sheaths, ligule, petiole, lamina, inflorescence, peduncle, thyrse, fertile bracts, coma bracts, cincinni, bracteoles, flowers, calyx, floral tube, corolla lobes, lateral staminodes, labellum, filament, anther, anther crest, stigma, ovary, fruit, and seeds. Colors were recorded from fresh material under natural daylight because some colors may change after preservation or drying. Morphological terminology generally follows Beentje [11].

### 2.3. Herbarium Study and Comparative Taxonomic Sources

A comparative taxonomic study was conducted using herbarium specimens, type material, protologues, original descriptions, subsequent taxonomic treatments, living collections, field observations, and high-resolution digital specimen images. Material available from the following herbaria and institutional collections was consulted either directly or through digital resources: A, AAU, B, BK, BKF, BM, BR, C, CAL, DMSC, E, HITBC, HN, HNPM, HNU, IBK, IMDY, K, KKU, L, NY, P, QBG, RAF, SGN, SING, TNS, US, VMSU, VNM, and VNMN. Herbarium acronyms follow the Index Herbariorum [12].

To evaluate the identity and taxonomic placement of the newly collected plants, comparisons were initially made with all currently recognized species of *Curcuma* subgen. *Hitcheniopsis* for which adequate morphological information was available, including material from Thailand and other countries. Attention was given to underground organs, lamina morphology, inflorescence architecture, fertile bract and coma bract morphology, flower exsertion, lateral staminodes, labellum morphology and colour pattern, indumentum, anther morphology, and anther-crest structure.

Detailed morphological comparison and phenetic analysis focused on ten comparative taxa selected according to three criteria: (1) placement within *Curcuma* subgen. *Hitcheniopsis*; (2) overall morphological similarity to, or potential confusion with, *C. koonmeeana* in vegetative, inflorescence, or floral characters; and (3) availability of sufficiently complete and comparable morphological information. The comparative set comprised *C. charanii*, *C. harmandii*, *C. parviflora*, *C. prasina*, *C. puangpeniae*, *C. purpurata*, *C. saraburiensis*, *C. thorelii*, *C. ubonensis*, and *C. × lopburiensis*. Together with *C. koonmeeana*, these constituted 11 operational taxonomic units (OTUs).

### 2.4. Phenetic Analysis

Phenetic relationships among the 11 taxa were evaluated at the taxon level, with each taxon treated as a single operational taxonomic unit (OTU). The 30 mature individuals examined for *C. koonmeeana* were used to document intraspecific variation and establish morphological ranges for the taxonomic description; they were not treated as separate OTUs because comparable individual-level datasets were unavailable for the other taxa.

The dataset comprised 34 morphological characters, including 24 quantitative and 10 qualitative characters (Appendix A.1 and Appendix A.2). Characters were selected to provide comparable representation of vegetative, inflorescence, floral, and androecial morphology while minimizing redundancy and excluding characters for which comparable information was incomplete or character states were strongly overlapping, transitional, or difficult to delimit consistently among taxa. Fruit, seed, and aril characters were excluded because comparable information was unavailable for several taxa.

For quantitative characters reported as ranges, a single taxon-level value was calculated as the midpoint of the normal reported range. Exceptional measurements presented in parentheses were excluded from midpoint calculations. This procedure was applied consistently to continuous measurements and numerical count characters. Qualitative characters were coded using predefined states representing broad, discrete, and comparable morphological conditions. The complete matrix comprised 34 characters × 11 taxa (374 data points).

Morphological dissimilarity among taxa was calculated using the Gower coefficient (Appendix A.3) [13]. The resulting Gower distance matrix was subjected to the unweighted pair-group method with arithmetic mean (UPGMA) clustering [14] and principal coordinates analysis (PCoA). The percentage of variation represented by each principal coordinate axis was calculated from the positive eigenvalues. Phenetic analyses were conducted using PAST version 4.15 [15]. The analyses were used to summarize overall morphological resemblance among the selected taxa rather than as an independent test of species boundaries.

### 2.5. Preliminary Conservation Assessment

The preliminary conservation status of the new species was evaluated following the Guidelines for Using the IUCN Red List Categories and Criteria [16]. The assessment considered the number and distribution of known populations, visually estimated population size, habitat condition, observed anthropogenic threats, and the availability of potentially suitable habitat.

The species is currently known from only two populations situated along the same mountain range. Population abundance was estimated visually during field surveys rather than through a complete census, and the exact number of mature individuals therefore remains uncertain. Particular attention was given to observed removal of plants from the wild for the ornamental trade as a potential continuing threat. Because systematic population censuses and comprehensive surveys of potentially suitable surrounding habitats have not yet been completed, the proposed conservation assessment is treated as preliminary pending additional field surveys and long-term population monitoring.

### 2.6. Nomenclature

The new species was prepared and described in accordance with the International Code of Nomenclature for algae, fungi, and plants (Madrid Code) [17]. The proposed name was checked against the International Plant Names Index (IPNI) [18] and Plants of the World Online (POWO) [1] to identify potential homonyms and to verify the accepted nomenclature of comparative taxa.

### 2.7. Figure Preparation

Photographic plates and figure compositions were prepared using Pixelmator Pro (version 3.6.5, “Archipelago”; Pixelmator Team, Vilnius, Lithuania) on a MacBook Pro (13-inch, M1, 2020; Apple Inc., Cupertino, CA, USA). Scientific illustrations were drawn by Thawatphong Boonma using an iPad Air (5th generation; Apple Inc., Cupertino, CA, USA) running iPadOS 17.5.1.

## 3. Results

The results of the taxonomic, morphological, phenetic, and conservation assessments are presented below. Detailed examination confirmed that the newly collected plants represent a morphologically distinct taxon within *Curcuma* subgen. *Hitcheniopsis*. The species is described and illustrated here, followed by comparisons with morphologically allied taxa and the results of the phenetic analysis based on 34 morphological characters. The recognition of this new species increases the known native diversity of subgen. *Hitcheniopsis* in Thailand to 31 taxa (comprising 30 species and one natural hybrid) [1,2,5,7,9,19].

### 3.1. Taxonomic Treatment of New Species

***Curcuma koonmeeana* Saensouk, P.Saensouk & Boonma, sp. nov.**

**Type:** THAILAND, Central, Saraburi Province, Phra Phutthabat District, c. 158 m elevation, 11 August 2025, *T.Boonma SB2581* (holotype VMSU).

**Diagnosis.** *Curcuma koonmeeana* is morphologically most similar to *C. charanii* in having short, non-creeping rhizome branches, glabrous laminae and fertile bracts, exserted flowers, and similarly coloured lateral staminodes, but differs in having an internally pale-yellow rhizome (vs. ivory), globose to subglobose tuberous roots (vs. fusiform), undulate lamina margins (vs. entire), fertile bracts with the lower ones obtrullate and the upper ones rhomboid to lanceolate (vs. broadly ovate), longer, well-exserted flowers (vs. exserted), narrower lateral staminodes with a broadly obtuse to occasionally acute apex (vs. broader, with a rounded apex), a larger, deeply bilobed labellum with a V-shaped sinus c. 8 mm deep (vs. emarginate, with a sinus 4–5 mm deep), and a white filament (vs. pale purple) (Table 1, Figure 2 and Figure 3).

**Description:** Perennial herb, 40–70 cm tall. Rhizome ovoid to subglobose, 1.5–2.2 × 1.4–2.0 cm, externally pale brown, internally pale yellow, fragrant; rhizome branches short, up to 3 cm long, c. 5 mm in diam., curved and producing the new shoot at the distal part; root tubers globose to subglobose, 1.2–1.8 × 1.2–1.8 cm, internally white, developing 6–8 cm away from the rhizome. Leafy shoot 40–70 cm tall, with 3–5 leaves; bladeless sheaths usually 3, 3–14 cm long, reddish brown or green with a reddish-brown tinge, decaying and becoming brown with age, glabrous; leaf sheaths distichous, 15–22 cm long, reddish brown, green distally, glabrous; ligule bilobed, 2–3 mm long, lobes rounded to truncate, glabrous; petiole canaliculate, 5–22 cm long, green, glabrous; lamina narrowly ovate to elliptic, 15–34 × 5–10 cm, adaxially green, abaxially pale green, glabrous on both surfaces, base rounded to cuneate, usually rounded in the lower leaves and cuneate in the upper leaves, apex acuminate and slightly mucronate, mucro 1–1.5 mm long, margin undulate, veins slightly plicate. Inflorescence terminal; peduncle slender, cylindrical, 10–20 cm long, 3–4 mm in diam., glabrous; thyrse cylindrical, 6–8.5 × 2.5–3 cm; fertile bracts 9–15, 2.0–3.0 × 1.3–2.4 cm, gradually diminishing in size towards the apex, lower bracts obtrullate, upper bracts rhomboid to lanceolate, each bract connate in the lower half of its length, pale green with a darker green distal portion, glabrous on both surfaces, in lateral view obliquely spreading at c. 20–30° from the rachis, distal portion reflexed, apex acute; cincinni with 4–5 flowers; coma bracts inconspicuous and poorly developed, 2–3, lanceolate, 1.7–3.2 cm long, connate in the lower one-fourth to one-fifth of their length, pale green with a darker green distal portion, glabrous on both surfaces, distal portion reflexed, apex acute, uppermost bract smallest and erect; bracteoles narrowly ovate to lanceolate, 8–10 × 4–6 mm, ivory, the largest with a greenish distal tinge, apex acute, glabrous. Flowers 3.7–4.0 cm long, well exserted from the bracts; ovary globose to subglobose, 1–1.5 × 1.0–1.4 mm, white, glabrous; epigynous glands absent; style very slender, 2.5–2.7 cm long; stigma infundibuliform, c. 0.8 × 0.7 mm, white, glabrous; calyx c. 6 mm long, apex tridentate, with a unilateral incision 1–2 mm long, white, glabrous; floral tube 2.2–2.4 cm long, white externally and internally, glabrous. Dorsal corolla lobe ovate-triangular, hooded, c. 6 × 3 mm, apex obtuse, white, glabrous; lateral corolla lobes ovate-triangular, hooded, c. 5 × 3 mm, apex obtuse, white, glabrous. Lateral staminodes asymmetrical, oblanceolate, 8.0–9.0 × 2.5–3.0 mm, apex broadly obtuse, occasionally acute, apical margin undulate, white, apically tinged with very pale purple, and with part of the yellow patch at the base extending from the basal part of the labellum, sparsely puberulous. Labellum deeply bilobed, 11.0–12.0 × 12.0–12.5 mm, c. 3 mm wide at base, with a V-shaped sinus extending to c. 8 mm, pale lavender to purple, densely pubescent; lobes narrowly obovate, apex broadly obtuse, outer margins undulate, sinus margins entire; two vivid yellow patches bordering the entrance to the floral tube; with or without a slender yellow median band originating at the base and extending along the median axis to the sinus. Stamen c. 5 mm long; filament flattened, c. 2 × 1.8 mm, white, sparsely puberulent; anther spurless, c. 3 × 2 mm, connective tissue white, puberulent; anther crest c. 1 mm long, white, apex broadly obtuse to rounded, reflexed; anther thecae c. 1.7 mm long. Fruit subglobose, cream, 6–8 × 6–8 mm, glabrous. Seeds pale brown, with white arils.

**Distribution and habitat:** *Curcuma koonmeeana* is currently known only from Phra Phutthabat District, Saraburi Province, Central Thailand, where two populations have been recorded along the same limestone mountain range at elevations of 158–165 m a.s.l. It grows in mixed deciduous forests on limestone hills, mainly on the forest floor among exposed limestone rocks and accumulated leaf litter, in relatively dense herbaceous understory vegetation. Plants occur locally in groups, often occupying small soil pockets and humus-rich substrates among limestone rocks. Other Zingiberaceae occurring in the surrounding forest include *Curcuma saraburiensis* Boonma & Saensouk, *Kaempferia saraburiensis* Picheans., and *Globba colpicola* K.Schum.

**Phenology:** Flowering was observed in July to August, and fruiting was observed in August. Further field observations are needed to determine the full flowering and fruiting periods.

**Etymology:** The specific epithet “*koonmeeana*” is named in honor of Mrs. Koonmee Saensouk, in recognition of her invaluable support and contributions behind the study of Zingiberaceae diversity in Thailand.

**Additional specimens examined:** THAILAND, Central, Saraburi Province, Phra Phutthabat District, c. 165 m elevation, 11 August 2025, *T.Boonma SB2582* (VMSU). Saraburi Province, Phra Phutthabat District, c. 158 m elevation, 8 August 2026, *T.Boonma SB2680* (VMSU).

**Utilization:** Plants are collected from the wild and sold for cultivation as ornamental pot plants. No other uses have been recorded.

**Comparative specimens examined:**

- *Curcuma charanii*—Central Thailand. Lop Buri, Sa bot, *T.Boonma 4* (KKU); Lop Buri, Sa bot, *T.Boonma LB028* (VMSU); Saraburi, Phra Phutthabat (cultivated), *T.Boonma SB0026* (VMSU); Nakhon Nayok, Ban Na (Cultivated), *T.Boonma NY041* (VMSU).
- *Curcuma harmandii*—Central Thailand. Saraburi, Wihan Daeng, *T.Boonma SB0027* (VMSU).
- *Curcuma parviflora*—Central Thailand. Lop Buri, Khok Samrong, *T.Boonma LB038* (VMSU); Saraburi, Wihan Daeng, *T.Boonma SB0032* (VMSU).
- *Curcuma prasina*—Northeastern Thailand. Sakon Nakhon, *T.Boonma 38* (VMSU); Central Thailand (cultivated), Nakhon Nayok, Ban Na, *T.Boonma NY066* (VMSU).
- *Curcuma puangpeniae*—Southwestern Thailand. Ratchaburi (cultivated), *T.Boonma 3* (KKU). Central Thailand. Lop Buri, Phra Phutthabat (cultivated), *T.Boonma LB043* (VMSU); Saraburi, Kaeng Khoi (cultivated), *T.Boonma SB0036* (VMSU); Nakhon Nayok, Ban Na (cultivated), *T.Boonma NY067* (VMSU).
- *Curcuma purpurata*—Eastern Thailand. Si Sa Ket, *T.Boonma 7* (KKU); Nakhon Nayok, Ban Na (cultivated), *T.Boonma NY068* (VMSU).
- *Curcuma saraburiensis*—Central Thailand. Saraburi, Phra Phutthabat, *T.Boonma 2* (BKF); Saraburi, Phra Phutthabat, *T.Boonma SB0046* (VMSU); Lop Buri, Khok Samrong, *T.Boonma LB055* (VMSU).
- *Curcuma thorelii*—Northeastern Thailand. Sakon Nakhon, *T.Boonma 1901* (VMSU); Central Thailand. Saraburi, Muak Lek, *T.Boonma SB0050* (VMSU).
- *Curcuma ubonensis*—Eastern Thailand, Ubon Ratchathani, Sirindhorn, *T.Boonma 902* (KKU); Buri Ram, Non Din Daeng, *T.Boonma and S.Phimpha BR153* (VMSU).
- *Curcuma* × *lopburiensis*—Central Thailand. Lop Buri, Khok Samrong, *T.Boonma LB035* (VMSU).

The following identification key was prepared to facilitate discrimination of *Curcuma koonmeeana* sp. nov. from selected morphologically similar taxa included in the comparative assessment. The key emphasizes readily observable vegetative, inflorescence, and floral characters that provide reliable separation among the taxa (Table 2).

### 3.2. Phenetic Similarity Analysis

The PCoA based on 34 morphological characters summarized the distribution of the 11 taxa in multivariate morphological space (Figure 4). The first two axes explained 42.665% and 19.986% of the total variation, respectively, accounting for 62.651% cumulatively. *Curcuma harmandii* was strongly separated toward the positive side of Axis 1, whereas *C. ubonensis*, *C. parviflora*, *C. puangpeniae*, and *C. prasina* were positioned on the negative side of this axis. *Curcuma saraburiensis* and *C. purpurata* were differentiated primarily along the positive side of Axis 2. The natural hybrid *C. × lopburiensis*, reported as a hybrid between *C. saraburiensis* and *C. parviflora* [19], occupied a relatively central position in the ordination, above *C. koonmeeana* along Axis 2. *Curcuma koonmeeana* itself was positioned near the centre of the ordination space, with *C. thorelii* occurring nearby, while *C. charanii* was displaced toward the negative side of Axis 2. The ordination illustrates the relative morphological positions and patterns of resemblance among the sampled taxa rather than providing an independent test of species boundaries.

The UPGMA analysis based on 34 morphological characters revealed a structured pattern of overall morphological similarity among the 11 taxa (Figure 5). *Curcuma parviflora* and *C. ubonensis* formed the closest pair, with *C. prasina* subsequently joining this cluster. The natural hybrid *C. × lopburiensis* was associated with this assemblage at a slightly greater distance. In a separate subcluster, *C. koonmeeana* grouped most closely with *C. charanii*, and *C. puangpeniae* subsequently joined this pair. These two major assemblages merged at a higher distance level, followed successively by *C. thorelii*, *C. purpurata*, and *C. saraburiensis*. *Curcuma harmandii* formed the most distant branch and was the most morphologically divergent taxon in the analysis. The cophenetic correlation coefficient of 0.9121 indicated a good representation of the original Gower distance matrix by the UPGMA dendrogram.

### 3.3. Preliminary Conservation Status

*Curcuma koonmeeana* is currently known from only two populations occurring within the same mountain range in Phra Phutthabat District, Saraburi Province, Central Thailand. Field observations indicate that the total number of mature individuals is unlikely to exceed c. 500, although a complete census of mature individuals was not undertaken. The species has a highly restricted known distribution and occurs in mixed deciduous forest. Evidence of wild collection for the ornamental trade was observed at the known sites, and continued removal of plants may result in a decline in the number of mature individuals.

Based on the currently available data, *C. koonmeeana* is provisionally assessed here as Vulnerable (VU D1) according to the IUCN Red List Categories and Criteria [16]. The visually estimated total population of fewer than c. 500 mature individuals falls below the threshold of 1000 mature individuals for criterion D1. Further field surveys are required to determine its full distribution, obtain a more precise estimate of the number of mature individuals, and calculate its extent of occurrence (EOO) and area of occupancy (AOO). Additional distributional and population data may allow reassessment under other IUCN criteria. Protection of the known populations, together with ex situ propagation and measures to reduce collection pressure, is recommended.

## 4. Discussion

The discovery of *Curcuma koonmeeana* demonstrates that botanical diversity may remain incompletely documented even in regions that have already been subjected to relatively extensive floristic investigation. Saraburi Province was previously surveyed for Zingiberaceae, but some mountainous localities could not be adequately explored because of accessibility and logistical constraints [8]. The subsequent discovery of *C. koonmeeana* during targeted revisits to such areas therefore illustrates the value of continued and spatially refined field exploration. This is particularly relevant to Zingiberaceae, in which seasonal emergence and relatively short flowering periods can limit detectability and make reliable identification difficult outside the reproductive season. The present finding consequently reinforces the need for repeated surveys across different years and flowering periods rather than considering previously surveyed areas botanically exhaustive.

The taxonomic evidence supports the placement of *C. koonmeeana* within *Curcuma* subgen. *Hitcheniopsis*. The absence of epigynous glands and the spurless anther conform to the principal morphological characteristics of the subgenus [3,4,5]. Within this framework, however, the new species possesses a combination of characters not encountered in the allied taxa examined. Particularly informative features are the positional differentiation and posture of the fertile bracts, the presence of inconspicuous coma bracts, the degree of flower exsertion, the morphology and indumentum of the labellum, its yellow marking pattern, and the configuration of the anther crest. Importantly, these characters are distributed across different floral and inflorescence structures and therefore provide a more robust basis for species delimitation than reliance on a single conspicuous feature. This is especially important in *Curcuma*, where similarities in general habit or floral coloration can mask substantial differences in structural morphology.

Among the species compared, *C. charanii* [9] remains the most informative taxon for direct diagnostic comparison. The two species share several conspicuous features, including glabrous laminae and fertile bracts, gullet-type flowers, pale-purple coloration of the lateral staminodes, paired yellow markings near the base of the labellum, and reflexed anther crests. Nevertheless, the similarities are accompanied by clear structural differences. Of particular diagnostic value is flower exsertion: in *C. koonmeeana*, the distal portion of the floral tube itself projects beyond the fertile bract, producing the well-exserted condition evident in lateral view, whereas in the exserted condition of *C. charanii*, the distal floral organs extend beyond the bract while the apex of the floral tube remains approximately level with its distal margin. When considered together with differences in fertile-bract architecture, coma-bract development, labellum size, depth of division and indumentum, yellow patterning, and anther-crest posture, this distinction supports the separation of the two taxa despite their superficial floral resemblance.

The Gower similarity matrix was consistent with the comparative taxonomic assessment in identifying *C. charanii* as the taxon showing the greatest overall morphological resemblance to *C. koonmeeana* (S = 0.7874). This similarity reflects the combined contribution of vegetative, inflorescence, and floral characters across the complete morphological dataset. Nevertheless, overall phenetic similarity should not be equated with taxonomic identity. The separation of *C. koonmeeana* from *C. charanii* is based on consistent structural differences, particularly in fertile-bract architecture, flower exsertion, labellum size and division, and other diagnostic characters documented in the comparative taxonomic assessment.

The ordination and clustering analyses provide complementary summaries of overall morphological resemblance among the sampled taxa. In the PCoA, *C. koonmeeana* occupied a relatively central position within the morphological space, whereas the UPGMA analysis grouped it most closely with *C. charanii*, in agreement with the pairwise Gower similarity matrix. Other taxa were distributed at varying distances within the ordination and clustering patterns, reflecting broader morphological heterogeneity within the sampled members of subgen. *Hitcheniopsis*. The Gower coefficient is particularly suitable for this dataset because it allows quantitative and qualitative morphological information to be integrated within the same analysis [13]. Because the phenetic analyses are based on the same morphological evidence used in comparative taxonomy, they are interpreted here as descriptive summaries of overall morphological relationships rather than as independent validation of species boundaries.

The recognition of *C. koonmeeana* also contributes to a broader understanding of diversity within subgen. *Hitcheniopsis* in Thailand. Its inclusion brings the currently recognized native diversity of the subgenus in the country to 31 taxa, comprising 30 species and one natural hybrid [1,2,5,7,9,19]. In combination with other recently documented members of the subgenus [5,6], this continuing increase suggests that species richness remains underestimated, particularly among taxa with narrow distributions or specialized habitat associations. The occurrence of *C. koonmeeana* in mixed deciduous forest on limestone hills also highlights the importance of small and heterogeneous limestone landscapes as reservoirs of localized plant diversity. Continued investigation of comparable habitats in Central Thailand may therefore reveal additional populations of this species or other insufficiently documented members of the subgenus.

Taxonomic recognition also has immediate conservation implications. *Curcuma koonmeeana* is presently known from only two populations situated along the same limestone mountain range, so its known distribution is geographically concentrated even though plants may be locally numerous. Such concentration can increase vulnerability because localized habitat alteration or sustained collection pressure may affect a substantial proportion of the known population. This concern is particularly relevant because plants are already being removed from the wild for sale as ornamentals. The preliminary assessment as Vulnerable under criterion D1 [16] should therefore be viewed as an initial evaluation based on the information presently available rather than as a definitive characterization of its long-term conservation status. Accurate counts of mature individuals, systematic searches of potentially suitable habitats, and calculation of EOO and AOO are needed to determine whether additional criteria may become applicable.

Conservation action should consequently address both the species and the habitat supporting it. Ex situ propagation may reduce pressure on wild plants and provide material for conservation purposes, but it cannot replace maintenance of natural populations and their ecological setting. Because the two known populations occur within the same mountain landscape and the surrounding forest also supports other native Zingiberaceae, area-based conservation could provide broader benefits than measures directed solely at *C. koonmeeana*. The site should therefore be evaluated for its potential suitability for recognition under the Other Effective Area-based Conservation Measures (OECMs) framework where applicable and provided that the formal requirements for such recognition are met [20]. Incorporating such an approach with habitat protection, monitoring, and measures to discourage unsustainable wild collection could contribute to the persistence of both *C. koonmeeana* and the associated native flora.

Several aspects of the species nevertheless remain insufficiently known. Its current distribution is based on surveys of a limited portion of the Phra Phutthabat landscape, its complete flowering and fruiting periods have not yet been established, and the visually estimated abundance has not been verified through a systematic census of mature individuals. In addition, the present taxonomic interpretation is based primarily on detailed comparative morphology, while the phenetic analyses provide a quantitative summary of overall morphological relationships among the sampled taxa. Because both approaches rely on morphological evidence, the phenetic results are interpreted as complementary descriptions of overall morphological resemblance rather than as independent validation of species boundaries. Future molecular phylogenetic analyses would provide valuable complementary evidence for further evaluating the taxonomic relationships, phylogenetic position, and evolutionary placement of *C. koonmeeana*.

Taken together, the discovery of *C. koonmeeana* has significance beyond the description of an additional species. It demonstrates how renewed exploration of incompletely surveyed habitats, detailed examination of living material, comparative taxonomy, and quantitative analysis of morphological variation can jointly reveal previously overlooked diversity. At the same time, its restricted known distribution and documented collection from the wild illustrate how taxonomic discovery and conservation assessment are closely linked. Continued botanical exploration of limestone landscapes in Central Thailand will therefore be important not only for refining the taxonomy and distribution of subgen. *Hitcheniopsis* but also for identifying habitats requiring conservation attention.

## 5. Conclusions

*Curcuma koonmeeana* is recognized as a distinct species of *Curcuma* subgen. *Hitcheniopsis* primarily on the basis of consistent diagnostic differences revealed through detailed comparative morphology. Phenetic analyses based on 34 morphological characters across 11 taxa provide a quantitative summary of overall morphological relationships within the sampled group, with *C. charanii* showing the greatest overall similarity to the new species. These analyses are interpreted as complementary descriptions of morphological resemblance rather than as independent validation of species boundaries. The recognition of *C. koonmeeana* increases the known native diversity of subgen. *Hitcheniopsis* in Thailand to 31 taxa, comprising 30 species and one natural hybrid. Its discovery in a previously insufficiently explored part of Saraburi Province further emphasizes the importance of continued botanical exploration in documenting localized plant diversity. Given its restricted known distribution and ongoing collection from the wild, continued field surveys, population monitoring, habitat protection, and ex situ propagation are important for refining its conservation assessment and supporting its long-term persistence in the wild.

## Figures and Tables

**Figure 1:**
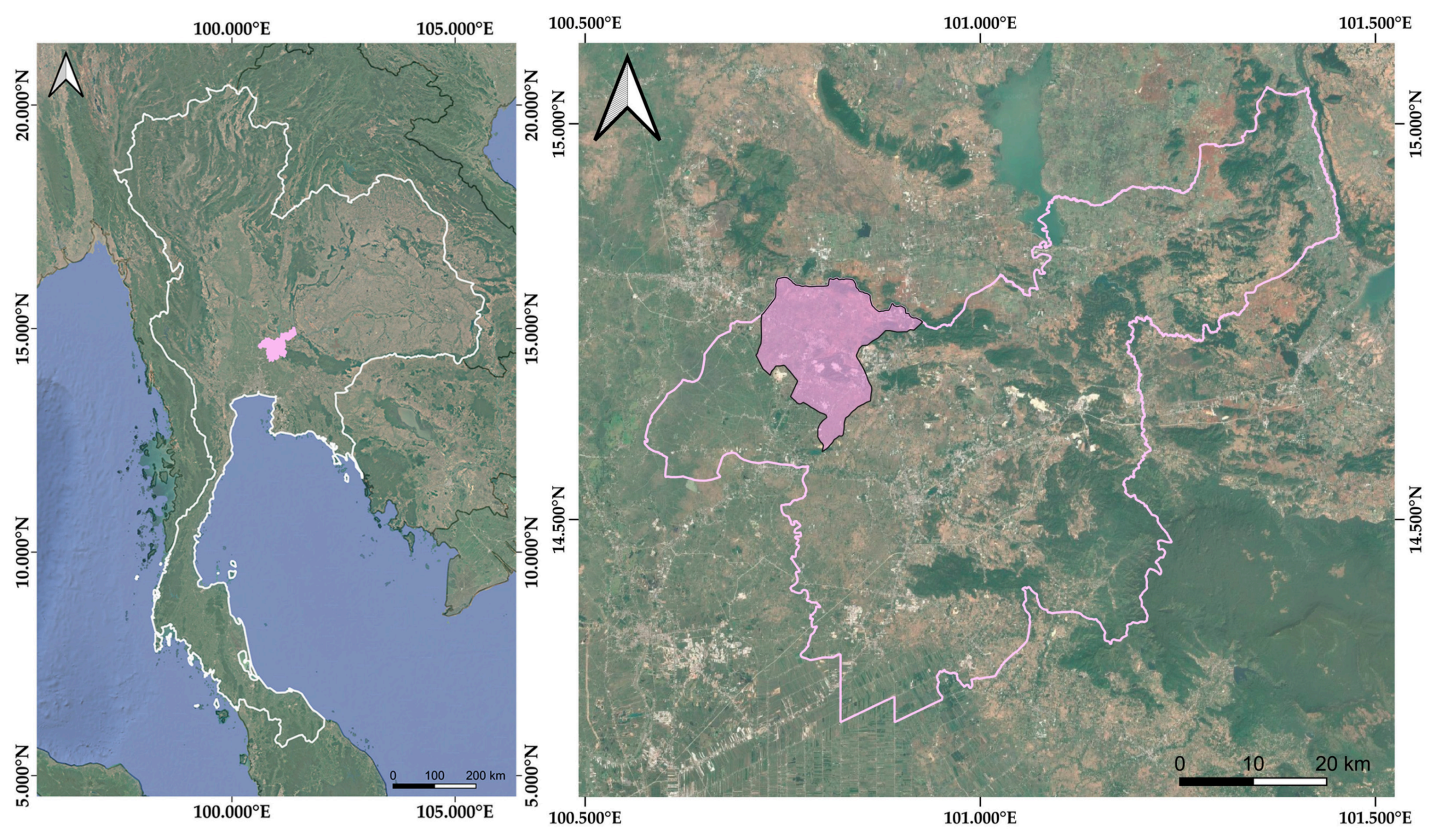
Location of the study area in Phra Phutthabat District, Saraburi Province, Central Thailand. The left panel shows the location of Saraburi Province within Thailand, and the right panel shows Phra Phutthabat District within Saraburi Province. Map created using QGIS version 3.34 with the WGS 84 geographic coordinate reference system (EPSG:4326). Map prepared by Kamonwan Koompoot.

**Figure 2:**
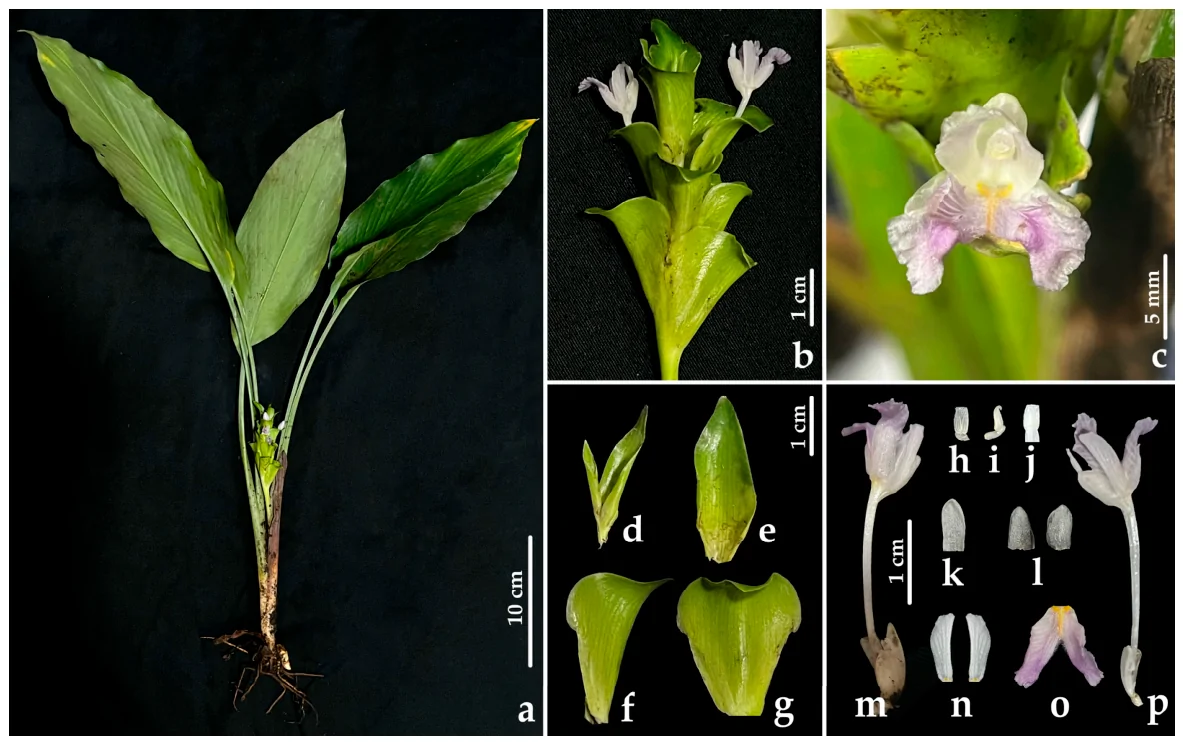
*Curcuma koonmeeana* Saensouk, P.Saensouk & Boonma, sp. nov.: (**a**) Habit. (**b**) Thyrse with flowers. (**c**) Front view of flower. (**d**,**e**) Coma bracts. (**f**) Lateral view of fertile bract. (**g**) Fertile bract. (**h**) Front view of anther. (**i**) Lateral view of anther. (**j**) Back view of anther. (**k**) Dorsal corolla lobe. (**l**) Lateral corolla lobes. (**m**) Cincinnus with bracteole and flower. (**n**) Lateral staminodes. (**o**) Labellum. (**p**) Flower with calyx and ovary. Photographs by Thawatphong Boonma.

**Figure 3:**
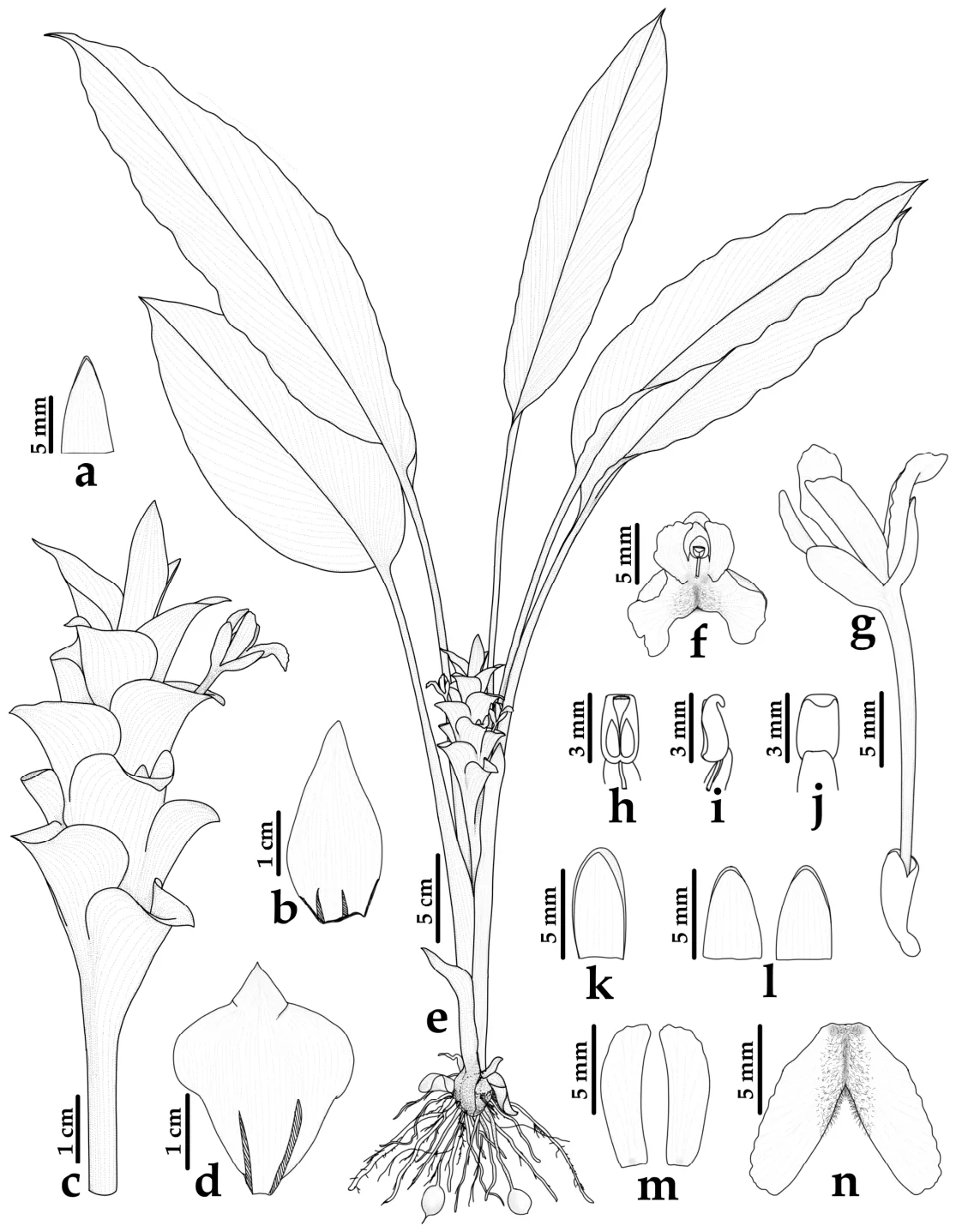
*Curcuma koonmeeana* Saensouk, P.Saensouk & Boonma, sp. nov.: (**a**) Bracteole. (**b**) Coma bract. (**c**) Thyrse with flower. (**d**) Fertile bract. (**e**) Habit. (**f**) Front view of flower. (**g**) Flower with calyx and ovary. (**h**) Front view of anther. (**i**) Lateral view of anther. (**j**) Back view of anther. (**k**) Dorsal corolla lobe. (**l**) Lateral corolla lobes. (**m**) Lateral staminodes. (**n**) Labellum. Drawn by Thawatphong Boonma.

**Figure 4:**
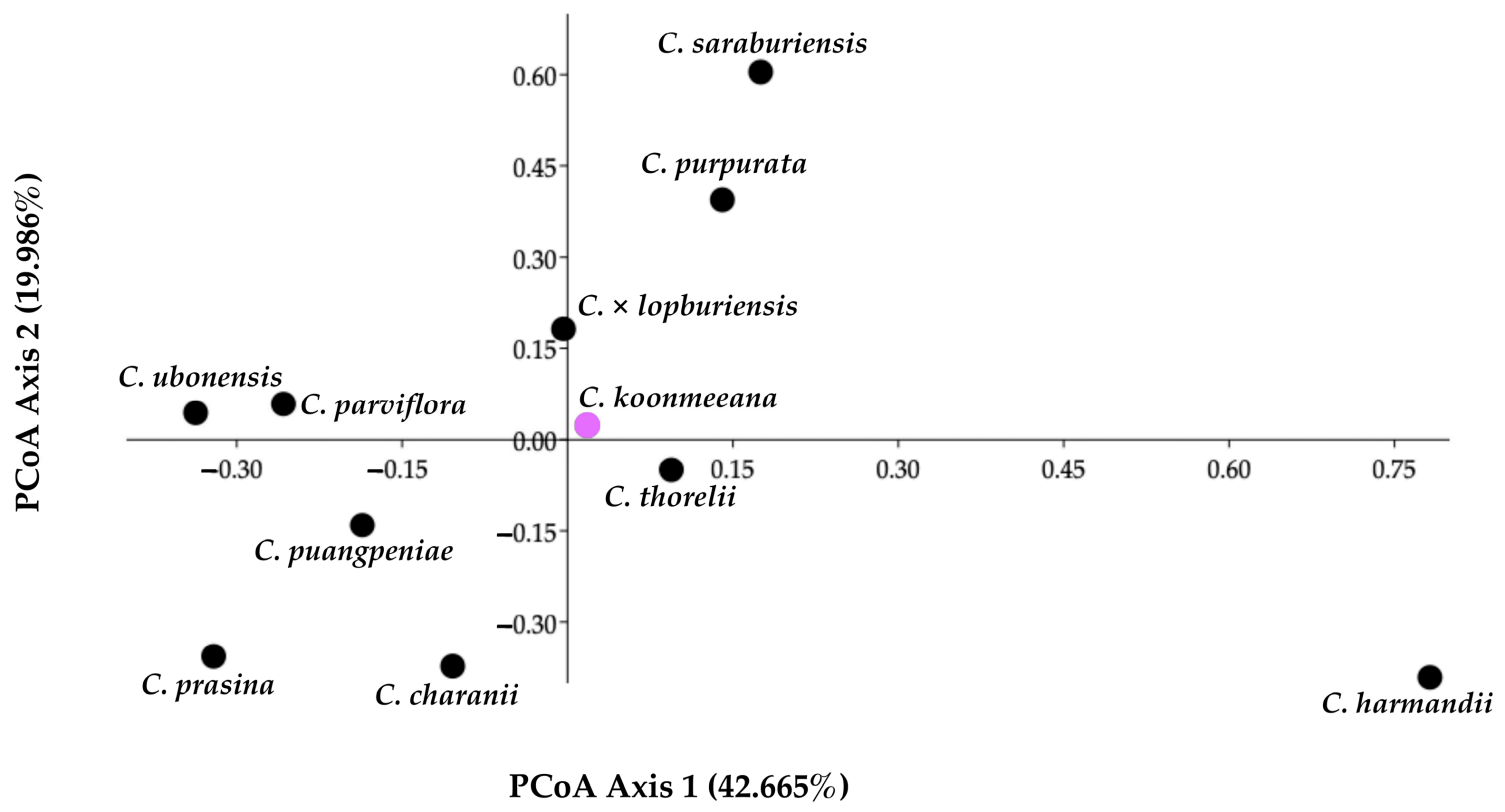
Principal coordinates analysis (PCoA) of *Curcuma koonmeeana* and ten morphologically allied taxa based on 34 morphological characters using the Gower coefficient. PCoA axes 1 and 2 explain 42.665% and 19.986% of the total variation, respectively. *Curcuma koonmeeana* is highlighted in purple.

**Figure 5:**
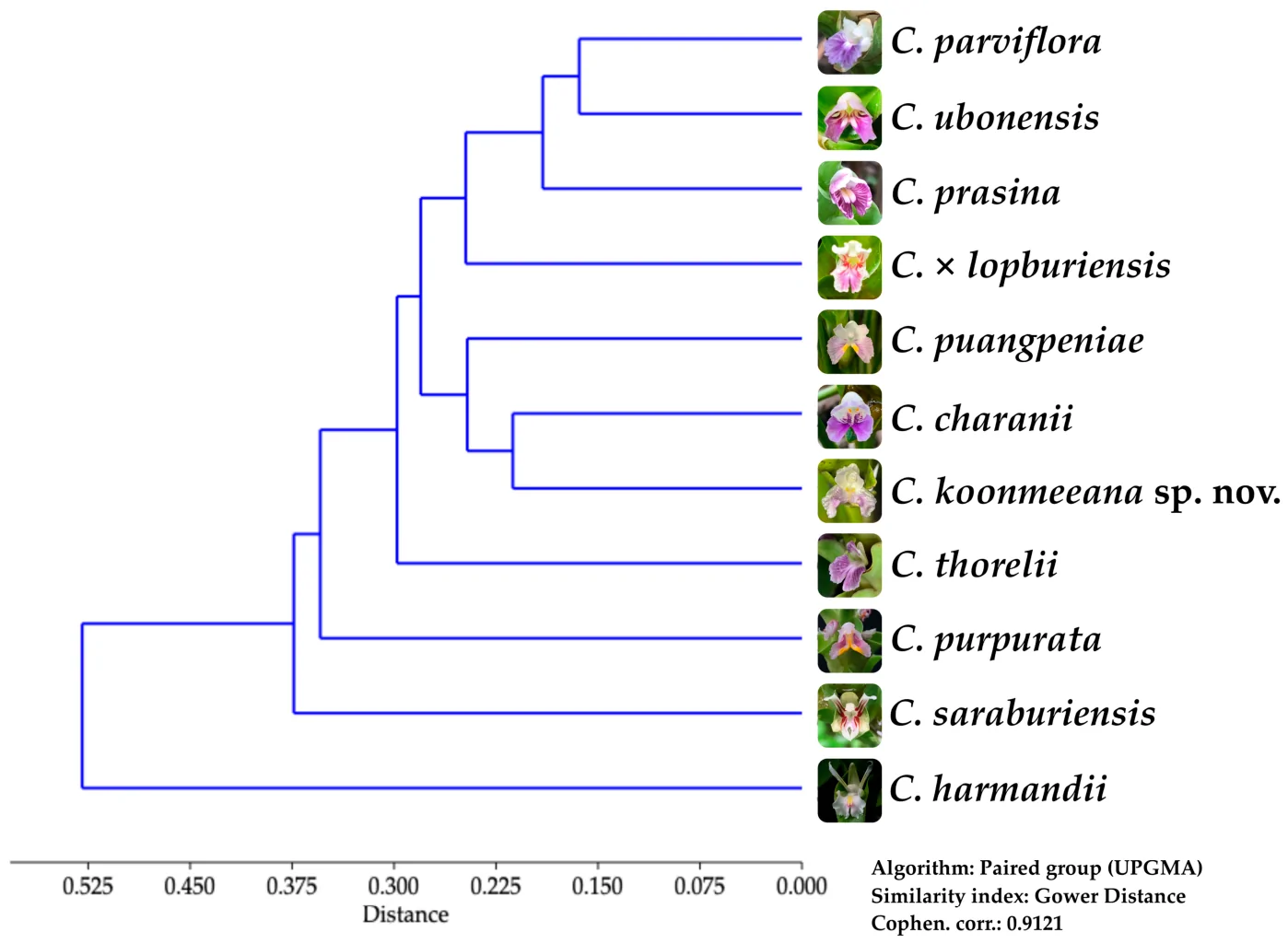
UPGMA dendrogram showing phenetic relationships among *Curcuma koonmeeana* and ten morphologically allied taxa based on 34 morphological characters using Gower distance. The cophenetic correlation coefficient was 0.9121, indicating a good representation of the original distance matrix by the dendrogram. Photographs illustrate the floral morphology of each taxon.

**Table 1:** Comparison of diagnostic morphological characters of *Curcuma koonmeeana* sp. nov. and the three most morphologically similar species.

Character	*C. koonmeeana* sp. nov.	*C. charanii*	*C. puangpeniae*	*C. purpurata*
**Internal rhizome color**	Pale yellow	Ivory	Pale yellow	Pale yellow
**Tuberous root shape**	Globose to subglobose	Fusiform	Ovoid	Ovoid to fusiform
**Rhizome branches**	Short, not creeping	Short, not creeping	Creeping	Short, not creeping
**Adaxial lamina surface**	Glabrous	Glabrous	Glabrous	Minutely pubescent
**Abaxial lamina surface**	Glabrous	Glabrous	Glabrous	Minutely pubescent
**Lamina margin**	Undulate	Entire	Entire	Undulate
**Thyrse**	6–8.5 × 2.5–3 cm	3.3–7 × 2.4–3.9 cm	3.8–6 (–7.2) × 2.5–3 (–4) cm	6.5–7.9 × 3.5–4.2 cm
**Fertile bracts number**	9–15	5–11	3–7	8–9
**Fertile bracts characters**	Lower ones obtrullate, upper ones rhomboid to lanceolate; obliquely spreading, distal portion reflexed	Broadly ovate, apex obtuse to acute	Broadly obovate, apex acute, tip curved outward	Asymmetrical obovate, apex obtuse with projecting dark-green tip
**Fertile bracts indumentum**	Glabrous	Glabrous	Glabrous	Minutely pubescent
**Coma bracts**	Present (2–3)	Absent	Absent	Present (2–3)
**Flowers**	3.7–4.0 cm long	3.5–3.8 cm long	3.0–3.2 cm long	c. 4.2 cm long
	Well exserted	Exserted	Exserted	Well exserted
**Lateral staminodes**	8–9 × 2.5–3 mm	7.3–8.7 × 3.7–4.4 mm	8.2–8.8 × 2.8–4.0 mm	c. 8.5 × 2.7 mm
**Lateral staminodes apex**	Broadly obtuse, occasionally acute	Rounded	Rounded to obtuse, sometimes retuse	Obtuse to acute
**Lateral staminodes color**	White, apically tinged very pale purple	White, apically tinged pale pinkish purple to pale purple	White	White
**Labellum**	Apex deeply bilobed	Apex bilobed	Apex bilobed	Apex deeply bilobed
	11–12 × 12–12.5 mm	9.8–11.2 × 8.6–9.5 mm	8–10 × 8–10 mm	9.5–9.8 × 8.9–9.4 mm
	Pale lavender to purple	Pale pinkish purple to purple	Lilac, fading to white basally	Purple basally, fading to pale purple distally
**Yellow markings on labellum**	Slender yellow median band expanding near the base into two conspicuous vivid-yellow patches	Small pale median line and two dark-yellow spots at base	Two short yellow dashes along each side of the incision; mid-lobe without a yellow median band	Yellow median bands extending along c. 2/3 of the incision, flanked by white stripes
**Labellum indumentum**	Densely pubescent	Finely glandular hairy medially	Glabrous	Hairy medially
**Labellum sinus depth**	c. 8 mm	4–5 mm	5–6 mm	7–8 mm
**Filament color**	White	Pale purple	White	White
**Anther crest**	c. 1 mm long	1–1.3 × c. 1.5 mm	c. 1 × 1.3 mm	c. 0.35 × 1.5 mm
	White	Very pale purple	White	White
	Reflexed	Slightly reflexed	Reflexed	Erect

**Table 2:** Key to *Curcuma koonmeeana* and selected morphologically similar taxa.

Characters	Species
1a. Fertile bract light green with pale green or white longitudinal lines and two white circular patches	2
1b. Fertile bract plain green, without white lines or patches	3
2a. Labellum margins of apical lobes entire	*C. saraburiensis*
2b. Labellum margins of apical lobes slightly crisped	*C.* × *lopburiensis*
3a. Abaxial surface of lamina pubescent	*C. purpurata*
3b. Abaxial surface of lamina glabrous	4
4a. Staminodes linear	*C. harmandii*
4b. Staminodes obovate to oblanceolate	5
5a. Margin of lamina red	*C. ubonensis*
5b. Margin of lamina green to white	6
6a. Coma bract well developed	7
6b. Coma bract absent or inconspicuous	8
7a. Lateral staminodes ovate oblong; coma bract white with green tip	*C. parviflora*
7b. Lateral staminodes obovate; coma bract pure white	*C. thorelii*
8a. Lamina width less than 3 cm	*C. prasina*
8b. Lamina width broader than 3 cm	9
9a. Labellum without two yellow patches at base but two short yellow dashes each side of the incision margin	*C. puangpeniae*
9b. Labellum with two yellow patches at base, without dashes	10
10a. Fertile bracts broadly ovate and essentially uniform in shape; flowers exserted, apex of floral tube approximately level with distal margin of fertile bract; labellum with sinus 4–5 mm deep	*C. charanii*
10b. Fertile bracts positionally differentiated, lower ones obtrullate and upper ones rhomboid to lanceolate; flowers well exserted, distal portion of floral tube projecting beyond fertile bract; labellum deeply bilobed with V-shaped sinus c. 8 mm deep	*C. koonmeeana*

## Data Availability

The morphological character definitions and coded character-state matrix used in the phenetic analyses are provided in Table A1 and Table A2. Additional data are available from the corresponding author upon reasonable request.

## Abbreviations

The following abbreviations are used in this manuscript:

A	Harvard University, Cambridge, Massachusetts, U.S.A.
AAU	Aarhus University, Denmark
B	Botanic Garden and Botanical Museum Berlin-Dahlem, Germany
BK	Bangkok Herbarium, Department of Agriculture, Thailand
BKF	Department of National Parks, Wildlife and Plant Conservation, Thailand
BM	The Natural History Museum, London, England, U.K.
BR	Meise Botanic Garden, Meise, Belgium
C	University of Copenhagen, Copenhagen, Denmark
CAL	Botanical Survey of India, Howrah, West Bengal, India
DMSC	Medicinal Plants Research Institute, Thailand
E	Royal Botanic Garden Edinburgh, Edinburgh, United Kingdom
HITBC	Xishuangbanna Tropical Botanical Garden, Academia Sinica, Yunnan, China
HN	Vietnam Academy of Science and Technology (VAST), Hanoi, Vietnam
HNPM	National Institute of Medicinal Materials, Vietnam
HNU	VNU University of Science, Hanoi, Vietnam
IBK	Guangxi Institute of Botany, China
IMDY	Chinese Academy of Medical Sciences, China
K	Royal Botanic Gardens, United Kingdom
KKU	Khon Kaen University Herbarium, Thailand
L	Naturalis Biodiversity Center, Leiden, Netherlands
NY	The New York Botanical Garden, New York, U.S.A.
P	Muséum National d’Histoire Naturelle, Paris, France
QBG	Queen Sirikit Botanic Garden, The Botanical Garden Organization, Thailand
RAF	Forest Research Institute, Yezin, Nay Pyi Taw, Myanmar
SGN	Southern Institute of Ecology, Ho Chi Minh City, Vietnam
SING	Singapore Botanic Gardens, National Parks Board, Singapore
TNS	National Museum of Nature and Science, Tokyo, Japan
US	Smithsonian Institution, Washington, U.S.A.
VMSU	Vascular Plant Herbarium, Mahasarakham University, Thailand
VNM	Institute of Life Science, Ho Chi Minh City, Vietnam
VNMN	Vietnam National Museum of Nature, Hanoi, Vietnam

## Appendix A

### Appendix A.1

The morphological characters used in the phenetic analysis were selected based on comparability among taxa, relative independence, completeness of available data, and the ability to define discrete and consistently applicable character states. Character definitions, coding schemes, and PAST data types are summarized in Table A1.

### Appendix A.2

The selected morphological characters were scored for *Curcuma koonmeeana* sp. nov. and ten morphologically allied taxa. The complete coded morphological matrix used in the phenetic analysis is presented in Table A2.

### Appendix A.3

Pairwise morphological distances among the 11 taxa were calculated using the Gower coefficient in PAST. For ease of interpretation, the resulting distances were converted to similarity values as *S* = 1 − *D*. The resulting Gower similarity matrix is presented in Table A3.

**Table A1:** Morphological characters and coding used in the phenetic analysis.

No.	Character	Character Type	Character ID	PAST Type	Unit/State Definition
1	Leafy shoot height	Vegetative	C01	Quantitative	cm
2	Number of leaves	Vegetative	C02	Quantitative	count
3	Ligule length	Leaf	C03	Quantitative	mm
4	Petiole length	Leaf	C04	Quantitative	cm
5	Lamina length	Leaf	C05	Quantitative	cm
6	Lamina width	Leaf	C06	Quantitative	cm
7	Peduncle length	Inflorescence	C07	Quantitative	cm
8	Thyrse length	Inflorescence	C08	Quantitative	cm
9	Thyrse width	Inflorescence	C09	Quantitative	cm
10	Number of fertile bracts	Inflorescence	C10	Quantitative	count
11	Fertile bract length	Bract	C11	Quantitative	cm
12	Fertile bract width	Bract	C12	Quantitative	cm
13	Flowers per cincinnus	Inflorescence	C13	Quantitative	count
14	Flower length	Floral	C14	Quantitative	cm
15	Calyx length	Floral	C15	Quantitative	mm
16	Floral tube length	Floral	C16	Quantitative	cm
17	Lateral staminode length	Floral	C17	Quantitative	mm
18	Lateral staminode width	Floral	C18	Quantitative	mm
19	Labellum length	Floral	C19	Quantitative	mm
20	Labellum width	Floral	C20	Quantitative	mm
21	Labellum sinus depth	Floral	C21	Quantitative	mm
22	Filament length	Androecium	C22	Quantitative	mm
23	Anther length	Androecium	C23	Quantitative	mm
24	Anther crest length	Androecium	C24	Quantitative	mm
25	Abaxial lamina indumentum	Leaf	C25	Binary	0 = glabrous; 1 = pubescent or minutely pubescent
26	Distal orientation of fertile bract	Bract	C26	Ordinal	0 = erect to ascending, distal portion neither spreading outward nor curved downward; 1 = spreading to subhorizontal, distal portion directed outward and approximately perpendicular to the inflorescence axis, but not curved downward; 2 = reflexed or curved downward, irrespective of the degree of curvature
27	Fertile bract connation	Bract	C27	Ordinal	0 = ≤1/4 of bract length; 1 = >1/4 to ≤1/3; 2 = >1/3 to ≤1/2; 3 = >1/2
28	Fertile bract indumentum	Bract	C28	Binary	0 = glabrous; 1 = pubescent or minutely pubescent
29	Coma bract development	Coma bract	C29	Ordinal	0 = absent; 1 = present but not forming a clearly differentiated terminal series; 2 = present as a distinct terminal series of sterile bracts differing from fertile bracts in shape and/or colour
30	Bracteole shape	Bracteole	C30	Nominal	0 = triangular; 1 = ovate to broadly ovate; 2 = narrowly ovate to lanceolate
31	Flower type	Floral	C31	Binary	0 = gullet type; 1 = open type
32	Flower exsertion	Floral	C32	Ordinal	0 = not or only slightly exserted, with the floral tube remaining enclosed within the fertile bract; 1 = exserted, with the apex of the floral tube approximately level with the distal margin of the fertile bract; 2 = well exserted, with the distal portion of the floral tube projecting beyond the fertile bract
33	Lateral staminode shape	Floral	C33	Nominal	0 = linear; 1 = ovate to oblong, including narrowly ovate–oblong; 2 = obovate to oblanceolate, including narrowly obovate, narrowly obovate–oblanceolate, and unequally obovate
34	Labellum indumentum	Floral	C34	Binary	0 = glabrous; 1 = hairy, puberulous, pubescent, or glandular-hairy, irrespective of distribution

**Table A2:** Coded morphological data matrix of Curcuma koonmeeana sp. nov. and ten comparative taxa used for phenetic analysis.

Taxon	*C. koonmeeana*	*C. charanii*	*C. harmandii*	*C. parviflora*	*C. prasina*	*C. puangpeniae*	*C. purpurata*	*C. saraburiensis*	*C. thorelii*	*C. ubonensis*	*C.* × *lopburiensis*
C01	55	24	50	32.5	27.5	35	40	40	70	32.5	40
C02	4	3.5	3.5	4	2.5	5	5	5.5	3.5	4	4
C03	2.5	1.5	1.5	1.5	1	2.5	2.5	3	1	1.25	2.5
C04	13.5	10	10.5	15.75	7	17	11	11	10	3.75	8
C05	24.5	15.25	22.5	16	15	17.5	26	19	25	18.5	19.5
C06	7.5	5.35	8.25	5.5	2.35	5.5	10.5	8	9.5	5.4	8.75
C07	15	8.6	27.5	10	13.5	14	20	22.5	20	8.35	15
C08	7.25	5.15	12	7	4.25	4.9	7.2	10	9.5	6.7	8
C09	2.75	3.15	7	3.5	2.75	2.75	3.85	3.5	4.5	3.25	3.25
C10	12	8	8	16	12	4	8.5	15	17.5	11.5	12
C11	2.5	2.35	7.25	2.25	1.8	2.1	2.845	2.25	3	3.25	2.5
C12	1.85	2.65	2.5	2.1	1.75	3.25	2.375	2.25	2.5	1.45	1.75
C13	4.5	7	12.5	3	4.5	3.5	3.5	5	6	4.5	5
C14	3.85	3.65	4.5	2.1	2.5	3.1	4.2	2.6	3	2.4	3.1
C15	6	5.6	11	5.5	5	4	8.4	16.5	5.5	7	9
C16	2.3	2.15	2.2	1.4	1.5	2.1	3	1.65	1.35	1.35	2
C17	8.5	8	11.5	6.75	7.5	8.5	8.5	14	12	7	12.5
C18	2.75	4.05	1.5	2.5	3.75	3.4	2.7	2.5	3	4	3.5
C19	11.5	10.5	17.5	9	8	9	9.65	9	13.5	7.25	12
C20	12.25	9.05	11	6.5	6	9	9.15	13	9	8.25	9
C21	8	5	7	1.5	3	5	5	9	5	3.75	5
C22	2	2	2.75	2	2	1.2	3.2	2.5	1.25	2	5
C23	3	3	6	3	3	2.65	3.5	5.5	3	2.2	4.5
C24	1	1.15	3.5	1.25	1.3	1	3.5	1.5	1.25	0.5	1.5
C25	0	0	0	0	0	0	1	1	0	0	0
C26	2	2	1	2	2	1	2	2	2	2	2
C27	2	2	0	2	2	2	2	2	2	2	2
C28	0	0	0	0	0	0	1	0	0	0	0
C29	1	0	0	2	0	0	2	2	2	2	2
C30	2	2	0	1	0	1	1	1	0	1	1
C31	0	0	1	0	0	0	0	1	0	0	0
C32	2	1	0	0	0	2	2	1	1	1	0
C33	2	2	0	1	2	2	2	1	2	2	1
C34	1	1	1	0	0	0	1	0	1	0	0

**Table A3:** Gower similarity matrix among Curcuma koonmeeana sp. nov. and ten comparative taxa based on 34 morphological characters.

Taxon	*C. koonmeeana*	*C. charanii*	*C. thorelii*	*C. purpurata*	*C. × lopburiensis*	*C. puangpeniae*	*C. ubonensis*	*C. parviflora*	*C. prasina*	*C. saraburiensis*	*C. harmandii*
*C. koonmeeana*	1.00000000	0.78735677	0.74500207	0.74458367	0.73482904	0.73300187	0.70668267	0.69249367	0.65759483	0.63694208	0.50690554
*C. charanii*	0.78735677	1.00000000	0.72385094	0.65880394	0.70259966	0.77470241	0.77526524	0.73520646	0.77920130	0.55565556	0.50724992
*C. thorelii*	0.74500207	0.72385094	1.00000000	0.68256295	0.73018955	0.62474021	0.71584887	0.70213668	0.67350814	0.63424960	0.55330751
*C. purpurata*	0.74458367	0.65880394	0.68256295	1.00000000	0.68145269	0.64496236	0.61847514	0.60645659	0.52832926	0.63982921	0.47826420
*C. × lopburiensis*	0.73482904	0.70259966	0.73018955	0.68145269	1.00000000	0.69333281	0.77102058	0.77086486	0.71618880	0.74099106	0.51878532
*C. puangpeniae*	0.73300187	0.77470241	0.62474021	0.64496236	0.69333281	1.00000000	0.72055322	0.71624599	0.72131358	0.58201034	0.43522694
*C. ubonensis*	0.70668267	0.77526524	0.71584887	0.61847514	0.77102058	0.72055322	1.00000000	0.83620580	0.81543439	0.63015936	0.37604341
*C. parviflora*	0.69249367	0.73520646	0.70213668	0.60645659	0.77086486	0.71624599	0.83620580	1.00000000	0.80328413	0.66764680	0.41467901
*C. prasina*	0.65759483	0.77920130	0.67350814	0.52832926	0.71618880	0.72131358	0.81543439	0.80328413	1.00000000	0.54861415	0.40897722
*C. saraburiensis*	0.63694208	0.55565556	0.63424960	0.63982921	0.74099106	0.58201034	0.63015936	0.66764680	0.54861415	1.00000000	0.50660817
*C. harmandii*	0.50690554	0.50724992	0.55330751	0.47826420	0.51878532	0.43522694	0.37604341	0.41467901	0.40897722	0.50660817	1.00000000

Note: Gower similarity values were calculated as *S* = 1 − *D*, where *D* is the Gower distance obtained from PAST.
